# Paediatric non-infectious uveitis in Cape Town, South Africa: a retrospective review of disease characteristics and outcomes on immunomodulating treatment

**DOI:** 10.1186/s12969-021-00537-x

**Published:** 2021-04-01

**Authors:** Waheba Slamang, Christopher Tinley, Nicola Brice, Christiaan Scott

**Affiliations:** 1 Department of Paediatric Rheumatology, Red Cross War Memorial Children’s Hospital, Klipfontein Rd, Cape Town, 7700 South Africa; 2 University of Cape Town South Africa, Rondebosch, Cape Town, South Africa; 3 Department of Paediatric Ophthalmology, Red Cross War Memorial Children’s Hospital, Klipfontein Rd, Cape Town, 7700 South Africa

**Keywords:** Non-infectious uveitis children juvenile idiopathic arthritis sub-Saharan Africa

## Abstract

**Background:**

Non-infectious uveitis is a well-reported cause of blindness in more developed countries, however data from sub-Saharan Africa is lacking. Here we aim to describe the diseases associated with paediatric non-infectious uveitis and the effect of currently available treatment in this setting.

**Methods:**

A retrospective observational analysis of children with non-infectious uveitis from January 2010 to December 2017, attending the tertiary paediatric rheumatology and ophthalmology referral units in Cape Town was conducted. Statistical analysis utilising STATA13 software was performed with *p* < 0.05 considered significant.

**Results:**

Twenty-nine children were identified: median age at first visit of 74 months (IQR 49–86 months), female to male ratio of 0.9:1, predominantly of mixed ancestry (72.4%). Juvenile idiopathic arthritis associated uveitis (JIAU) (48.3%), idiopathic uveitis (41.4%), sarcoidosis (6.9%) and Behcet’s disease (3.5%) were diagnosed. Chronic anterior uveitis (72.4%) was the most frequent finding. Fifty-five percent had complications at presentation and all children with idiopathic uveitis presented with cataracts.

Only 6.5% of the JIA cohort had JIAU. All JIA children had chronic anterior uveitis. There were no differences between JIA children with uveitis and those without uveitis, for sex (*p* = 0.68) and race (*p* = 0.58). Significantly, children with uveitis presented at an overall younger age (*p* = 0.008), had oligo-articular JIA (*p* = 0.01) and were antinuclear antibody positive (*p* < 0.001).

Children with idiopathic uveitis were predominantly male (66.6%) with chronic anterior uveitis (41.7%).

Nineteen children (65.5%) in the cohort had inactive disease on treatment at 12 months from diagnosis, which included 10 on topical corticosteroid therapy. At the last clinical visit 17 (58.6%) on standard initial therapy, 8 (27.6%) on tumour necrosis factor inhibitors and 2 on additional DMARDs were in remission. Five of these children still required topical corticosteroids. Surgery was performed in 41.4%, primarily in the idiopathic group. Visual acuity improved or was maintained on treatment.

**Conclusion:**

Current practice seems to detect children with potentially sight-threatening disease but the high rate of complications and the low percentage of children with JIAU raises concerns of delayed healthcare intervention. Tumour necrosis factor inhibitors have improved outcomes in refractory cases in this cohort, however further studies are needed.

## Background

Uveitis broadly describes inflammation of the iris, choroid and retina, which occurs when the blood-aqueous and blood-retinal barrier is disrupted. This may be triggered by infectious or non-infectious diseases and is an important cause of 16–25% of blindness worldwide. The estimated paediatric prevalence of 28:100000, is at least 4 times lower than in adults [1]. However, the sight-threatening consequences due to late presentation and aggressive disease are far-reaching in children, particularly in developing countries where employment opportunities for the visually impaired are limited [2].

Epidemiology studies highlight the paucity of data from Africa and other developing countries, noting potential differences in the prevalence and demographics of underlying aetiologies as well as in outcomes [1, 3]. In sub-Saharan African countries (SSA), uveitis is under-reported in surveys or studies on blindness in specific regions. In general, active uveitis resulting in visual loss tends to be documented and outcomes, rather than the underlying diseases are recorded [4–8]. Additionally, studies predominantly describe adult populations and infectious diseases [9–12]. In children, post-streptococcal syndrome and HIV-associated uveitis have also recently been described [13–15]. Idiopathic uveitis is diagnosed where an underlying systemic cause cannot be found despite diagnostic ocular paracentesis in some cases. This frequently presents with complications and still accounts for up to 50% of uveitis populations seen at referral centres [16–18].

The prevalence of paediatric immune-mediated systemic diseases and the associated uveitis, varies by underlying disease, disease subtype, as well as geographically [1]. In SSA, the limited data in children, describe the association with juvenile idiopathic arthritis (JIA). Notably BD is reported more frequently from countries around the Mediterranean basin and the Far East [1, 19–26]. In European and western countries, where the highest prevalence of JIA is reported, chronic anterior uveitis occurs in up to 20% of children diagnosed with the oligo-articular JIA subtype. These are predominantly ANA positive, young girls under 7 years old of Caucasian ancestry although recently, associations with age and sex have been questioned [27, 28]. Ancestry as a predictor of more aggressive disease and poorer outcomes has also been described but research in this area is ongoing [29]. While chronic anterior uveitis is associated with the psoriatic JIA subtype, acute anterior uveitis may also be seen in those presenting at an older age with an enthesitis-related arthritis phenotype [30, 31]. However, acute anterior uveitis is more commonly associated with enthesitis-related arthritis and psoriatic JIA, as well as in other HLA B27 associated diseases [27, 32].

The potential risk of amblyopia and poor long-term outcomes secondary to persistent disease activity and prolonged corticosteroid treatment in children is recognized. The standard uveitis nomenclature (SUN) working group classification and screening guidelines, though not formally validated in children, has improved cross-study comparisons. This has aided monitoring of treatment responses and decisions to escalate therapy. Informed by ongoing studies, treatment recommendations are further elaborated in recent management guidelines. [33–46].

Understanding the underlying causes of non-infectious, immune-mediated uveitis is essential for appropriate management and to improve overall visual outcomes. However, to date there are no studies reviewing non-infectious uveitis per se, in children from SSA.

### Aim

Here we aim to describe the disease characteristics and outcomes on immunomodulatory treatment, of children with non-infectious uveitis managed at a tertiary paediatric referral centre in Cape Town, South Africa.

## Methods

### Study design

A retrospective case file review of all children ≤16 years managed for non-infectious uveitis by the paediatric ophthalmology and rheumatology units in Cape Town from 1 January 2010 to 31 December 2017 was conducted.

The tertiary paediatric rheumatology and ophthalmology referral units at Groote Schuur and Red Cross Children’s Hospitals in Cape Town are the main tertiary referral centres for the Western Cape as well as other provinces in South Africa. The Western Cape population numbers around 6.2 million and children <15years constitute 26% [47]. For the purposes of this study, self-reported ancestry is considered, as associations with potentially higher risk and poorer outcomes have been described [28, 29].

### Data collection

Children with non-infectious uveitis managed by the tertiary referral paediatric ophthalmology and rheumatology units were included. Children with JIA were included if uveitis was found on screening during the study period. Screening for JIAU was performed at diagnosis and at 3 monthly intervals as per the Standard Uveitis Nomenclature (SUN) working group recommendations [34, 48]. Children were identified from a review of paediatric rheumatology (PR) and ophthalmology case files and confirmed on the hospital electronic booking system. Data of patients identified with non-infectious uveitis included age, sex, self-reported ancestry, date of first presentation, anatomical location of uveitis, visual acuity, (VA) and complications. For uniformity, VA was converted from the recorded annotation to the log of the minimal angle of resolution (LogMAR) [49]. Data of children with JIA additionally included JIA subtype, time to uveitis diagnosis and arthritis disease activity on treatment.

JIA was defined as per International League of Associations for Rheumatology criteria [50]. Idiopathic uveitis was diagnosed after exclusion of infective and other immune mediated diseases by clinical assessment and laboratory tests. These included but were not limited to toxocara and toxoplasma serology, HIV Elisa or polymerase chain reaction (PCR), ANA, anti-double stranded DNA, anti-streptolysin O (ASO) and, anti-deoxyribonuclease B (Anti-DNAse B) titres, serum angiotensin converting enzyme, Treponema Pallidum Haemagglutination test and/or Venereal Disease Research Laboratory test and urine dipstix. Ebstein barr virus, cytomegalovirus and Lyme disease (not endemic in the Western Cape region of South Africa) serology, were not routinely requested but may have been performed in individual cases**.** Sarcoidosis was diagnosed based on clinical presentation, histology +/− raised serum angiotensin levels and Behcet’s disease by clinical diagnosis as per the paediatric BD criteria 2015 [51].

Treatment modalities were grouped as standard initial treatment which included corticosteroids (topical and/or systemic) and methotrexate 10–20 mg/m^2^ (maximum dose 20 mg orally or 25 mg subcutaneously). Additional disease modifying anti-rheumatic drug therapy included azathioprine (1–3 mg/kg) and mycophenolate mofetil (250–500 mg/m^2^ bd). Biologic therapy included TNFi Infliximab 6–10 mg/kg iv infusion monthly (after loading) and Adalimumab 20–40 mg subcutaneously every second week.

The primary outcome was considered as clinically inactive disease on treatment. Ophthalmology assessments were performed at weekly to 3-monthly intervals depending on severity of disease. Uveitis activity at 12 months and at the last clinical visit was evaluated. Anterior chamber disease, vitreous haze and posterior disease were assessed utilising the SUN criteria [34]: The *response* to treatment was defined as a two-step decrease in inflammation or decrease to Grade 0, *active disease* as ≥ Grade 1 (6–15 cells/slit lamp field and faint anterior chamber flare), *inactive disease* as Grade 0 (< 1 cell/slit lamp field and no anterior chamber flare) and *worsening* of disease activity as a two-step increase in level of inflammation or an increase from 3+ to 4+ for anterior chamber disease. Vitreous haze was further evaluated by the Nussenblatt 1985 criteria [52] and posterior disease activity by resolution or non-resolution of retinal vasculitis and/or disc swelling.

For this study, remission was defined as ≥3 months of inactive disease on treatment.

The secondary outcome was considered as improvement in visual acuity.

Children managed for < 3 months or were lost to follow-up, as well as children with proven infectious uveitis were excluded from the study.

### Statistical analysis

Statistical analysis was performed using STATA13 software.

The frequencies of categorical variables were noted and descriptive statistics employed to determine measures of central tendency. Chi-squared (or Fisher exact tests if frequencies were < 5) and t-tests (or Wilcoxon sum rank tests for non-parametric data) for comparisons between groups, were used as appropriate to evaluate associations with *p* < 0.05 considered significant. Odds ratios for statistically significant variables were calculated to evaluate associated risk. Cox regression models was used to assess time to inactive disease and time to uveitis from JIA diagnosis.

## Results

Thirty-four children were referred for the management of uveitis. 19 (55.9%) presented to ophthalmology and 15 (44.1%) to rheumatology. Five with underlying infectious causes were excluded, resulting in 29 children meeting inclusion criteria. The overall group had a 0.9:1 female to male ratio, median age at first visit of 74 months (IQR 49–86 months) and were predominantly of mixed ancestry (72.4%). Children with JIAU (48.3%) and idiopathic uveitis (41.4%) most commonly had bilateral, chronic anterior uveitis (72.4%). Panuveitis (33.4%), acute anterior (6.9%) and intermediate uveitis (3.5%) were less frequent. All children with panuveitis had inflammation of the anterior chamber and vitreous, with retinal vasculitis and/or disc swelling. Fifty-five percent had cataracts at presentation (Table 1).

**Table 1:** Disease characteristics

Diagnosis	JIAU	Idiopathic	Sarcoidosis	Behcet’s Disease
Total N (%) (CI: 1.4–2.0)	*N* = 14 (48.3)	*N* = 12 (41.3)	*N* = 2 (6.9)	*N* = 1 (3.5)
No. of affected eyes (CI: 1.6–1.9)	26	20	4	1
Demographics				
Presentation age (months)				
Median (IQR) (CI: 62.6–87.8)	55 (34–86)	76.5 (67–85)	102.5 (49–156)	120 (-)
Female N (%) (CI: 1.3–1.7)	8 (57.1)	4 (33.3)	2 (100)	0
Ancestry N (%) (CI: 1.1–1.6)				
Mixed	10 (71.4)	9 (75)	1 (50)	1 (100)
Black African	2 (14.3)	2 (16.7)	1 (50)	0
Caucasian	2 (14.3)	1 (8.3)	0	0
Anatomical Location (CI: 1.3–2.2)				
Acute Anterior	0	1	1	0
Chronic Anterior	14	6	0	1
Intermediate	0	1	0	0
Posterior	0	0	1	0
Panuveitis	0	4	0	0
Laterality N (%)				
Unilateral	2	4	0	1
Bilateral	12	8	2	0
Complications (CI 1.0–1.9)				
No. affected children (N)	7	9	0	0
Cataracts	5	9	0	0
Posterior synechiae	3	3	0	0
Band Keratopathy	2	2	0	0
Raised intra- ocular pressure	3	4	0	0
VA at presentation (Median)				
LogMAR (IQR) (IQR)	0.1 (0.0–0.3)	0.95 (0.55–2.45)	0.0 (0.0–0.0)	0.3 (−)
VA at last visit				
Median LogMAR (IQR)	0 (0.0–0.2)	0.3 (0.0–0.6)	0 (0–0)	0.1 (−)
Change in VA: *p*-value	0.06	0.001	–	–

***JIAU*** Juvenile Idiopathic Arthritis associated Uveitis, ***CI*** 95% confidence interval, ***VA*** Visual Acuity, ***LogMAR*** Log of minimal angle of resolution, where LogMAR 0.0 ~ Snellen 6/ 6, LogMAR 0.5 ~ Snellen 6/19 and LogMAR 1.0 ~ Snellen 6/60

**Fig. 1:**
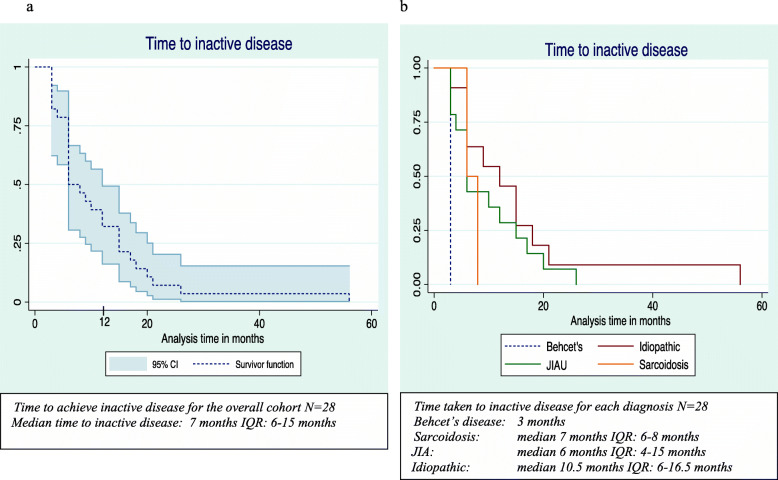
**a**: Time to inactive disease. **b**: Time to inactive disease by diagnosis

**Fig. 2:**
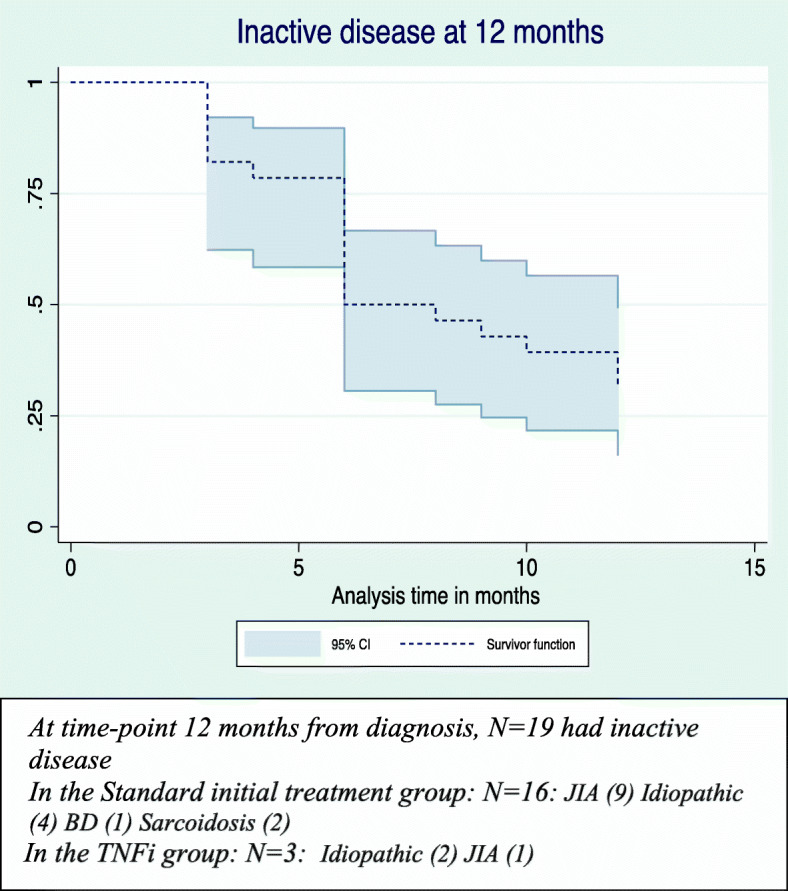
Inactive disease at 12 months

For the cohort, the median time from diagnosis to inactive disease was 7 months (IQR 6–15 months) (Fig. 1a). Although the median time varied depending on the underlying disease, there was no statistical difference (*p* = 0.28) between children with JIAU and idiopathic uveitis for overall time to inactive disease from diagnosis (Fig. 1b). Twenty children were diagnosed before 2017. Of these, 19 children had inactive disease on treatment at time-point 12 months after diagnosis, including 3 who had been started on TNFi (Fig. 2). 8 children in the standard treatment group and 1 on TNFi, no longer required topical steroid therapy. At the last clinical visit, 27 (93.1%) children were in remission. One (3.5%) had ongoing refractory disease and 1 (3.5%) had clinically inactive disease for < 3 months (Table 2). Twenty-two children no longer required topical steroid therapy. However, 2 children recently diagnosed with JIAU in the standard treatment group and 3 with idiopathic uveitis in the TNFi group, still required topical steroid therapy.

**Table 2:** Treatment

Treatment	Total treated N	Response (R) / No Response (NR)	JIAU *N* = 14		Idiopathic *N* = 12	Sarcoidosis *N* = 2	Behcet’s Disease *N* = 1	Total N (%)
			Screening *N* = 11	Pre-JIA diagnosis *N* = 3				
Standard Initial Rx	29	R	7	1	6	2	1	17 (58.6)
		NR	4	2	6	0	0	12 (41.4)
Azathioprine	8	R	1	–	0	–	–	1 (12.5)
		NR	3	–	4	–	–	7 (87.5)
Mycophenolate Mofetil	3	R	1	–	0	–	–	1 (33.3)
		NR	1	–	1	–	–	2 (66.6)
Infliximab	6	R	1	0	1	–	–	2 (33.3)
		NR	1	1	1	–	–	3 (50)
Adalimumab	8	R	1	2	4	–	–	7 (87.5)
		NR	0	0	1	–	–	1 (12.5)
Surgery: no. of children N (%)			2	1	9	0	0	12 (41.4)
No. of procedures								
Cataract incl. Pars planar vitrectomy/ lensectomy			2	1	10	0	0	13
Corneal chelation			0	0	2	0	0	2
Evisceration			0	0	1	0	0	1

Responders (R) had inactive disease on treatment. In non-responders (NR), treatment was escalated to one of the additional DMARDs then a TNFi or directly to a TNFi

There was an overall clinical improvement in visual acuity (VA) (Table 1).

### Disease characteristics

Children with JIAU were most commonly female (female to male ratio of 1:0.75), had a median age of 55 months (IQR 34–86 months), and were predominantly of mixed ancestry (71.4%). All children had chronic anterior uveitis (100%).

**Fig. 3:**
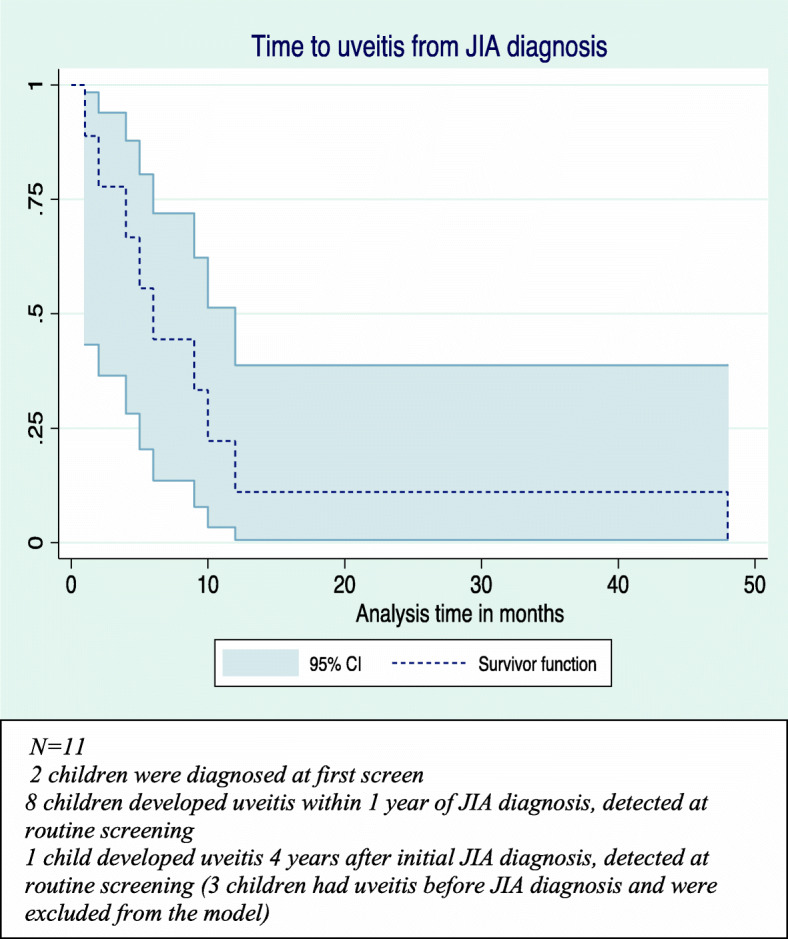
Time to uveitis from JIA diagnosis

Further analysis in relation to the overall JIA cohort managed during the study period was undertaken (Table 3). A total of 229 patients were assessed for JIA of which 12 were excluded according to ILAR criteria. The remaining 217 were evaluated, with a consequent JIAU clinic prevalence of 6.5%. Three children had uveitis for 12, 9 and 4 months prior to the diagnosis of JIA. The majority developed uveitis within a year of diagnosis (Fig. 3). Comparisons for sex (*p* = 0.68) and ancestry (*p* = 0.58) were not statistically significant. Children older than 144 months were not diagnosed (*p* = 0.01) with uveitis. Young age ≤ 84 months (*p* = 0.01), oligo-JIA subtype (*p* = 0.01) and positive ANA (*p* < 0.001) were significant factors. Univariate analysis showed odds ratios (OR) for possible risk factors associated with uveitis as oligo-articular subtype (OR 4.45 CI 1.35–14.7) and positive ANA (OR 33.3, CI 6.83–162.09). Seven (50%) children had complications. Reduced VA at presentation was due to cataracts in 5 children. There was no statistical difference in complications between those diagnosed with uveitis before arthritis and those with uveitis on screening at diagnosis and subsequently (*p* = 0.28). At time-point 12 months from diagnosis, 9 (64.3%) children on standard initial treatment and one on TNFi had inactive disease. By the end of the study period, 8 (57.1%) were in remission on standard initial treatment, 2 (14.3%) on additional DMARDs and the previously refractory 4 (28.6%) on TNFi treatment. Of children treated with TNFi, 3 were originally commenced on infliximab and 2 were switched to adalimumab due to lack of efficacy. Of these, one was diagnosed with uveitis 12 months before arthritis. The one child on infliximab and all children in the adalimumab group achieved remission. Three children had cataract surgery.

**Table 3:** Comparison of JIA with no uveitis and with uveitis (JIAU)

Diagnosis	JIA Total ***N*** = 217	JIA no uveitis ***N*** = 203	JIAU ***N*** = 14	***p***-value	
**Median Age** months (IQR)	111 (54–148)	114.5 (59–152)	55 (34–86)	†*p* = 0.008	
**Age groups (months) N (%)**					**Odds Ratio 95% CI**
0–84	70 (32.3)	60 (29.6)	10 (71.4)	†*p* = 0.01	0.46 CI 0.25–0.83
85–144	73 (33.6)	69 (33.9)	4 (28.6)	*p* = 0.78	0.71 CI 0.22–2.35
145–192	60 (27.7)	60 (29.6)	0	†*p* = 0.01	1.0 CI 0.6–1.7
**Female N (%)**	135 (62.2)	127 (62.5)	8 (57.1)	*p* = 0.68	0.78 CI 0.26–2.34
**Ancestry N (%)**					
Mixed	124 (57.1)	114 (56.2)	10 (71.4)	*p* = 0.40	0.72 CI 0.39–1.3
Black African	37 (17.1)	35 (17.2)	2 (14.3)	*p* = 1.00	1.25 CI 0.27–5.8
Caucasian	56 (25.8)	54 (26.6)	2 (14.3)	*p* = 0.53	0.46 CI 0.10–2.1
**JIA subtype N (%)**					
Oligo-articular	83 (38.3)	73 (35.9)	10 (71.5)	†*p* = 0.01	4.45 CI 1.35–14.7
Poly articular RF +	18 (8.3)	18 (8.8)	0	*p* = 0.61	0.08 CI 0.04–0.13
Poly articular RF –	43 (19.8)	40 (19.7)	3	*p* = 1.00	1.05 CI 0.54–2.04
Psoriatic	16 (7.4)	15 (7.4)	1	*p* = 1.00	0.99 CI 0.59–1.66
Systemic Onset JIA	17 (7.8)	17 (8.4)	0	*p* = 0.61	–
Enthesitis Related Arthritis	38 (17.5)	38 (18.7)	0	*p* = 0.14	–
Undifferentiated	2 (0.9)	2 (1)	0	*p* = 1.00	–
**Lab Parameters N (%)**					
ANA tested	147 (67.7)	136 (67)	11 (78.6)	†*p* < 0.001	33.29 CI 6.83–162.09
Hep2 Positive	15	10	5		
Elisa Positive	15	9	6		
RF tested	125 (57.6)	116 (57.1)	9 (64.3)	*p* = 0.36	–
Negative	107	98	9		
Positive	18	18	0		
HLA B27 tested Present	63 (29) 4	62 (30.5) 4	1 (7.1) 0	*p* = 1.00	–

Idiopathic uveitis (41.4%) was the next frequent diagnosis, presenting in males (66.6%), at a median age of 76.5 months (IQR 49–156 months) who were of of mixed ancestry (75%). Both eyes were affected in 66.6% with chronic anterior uveitis (50%). All children presented with cataracts and had a median VA LogMAR 0.95 (0.55–2.45). Four children (33.3%) had inactive disease on standard initial treatment and 2 (16.7%) on TNFi at 12 months. At the last clinical visit, 6 (50%) were in remission on standard initial treatment, 1 (8.3%) on infliximab and 4 (33.3%) on adalimumab. 75% of children required cataract surgery. One child did not respond to therapy, was ANA positive and needed evisceration of one eye due to painful glaucoma. The overall improvement in VA for the remaining children was statistically significant (*p* = 0.001).

Sarcoidosis (6.9%) was diagnosed in 2 females. Both had bilateral uveitis and no complications at presentation. They had inactive disease by 12 months, achieved remission on standard initial therapy and had preserved vision.

The 120 month old male of mixed ancestry with BD, presented with unilateral chronic anterior uveitis, had no complications and responded to standard therapy within 3 months.

## Discussion

Non-infectious immune-mediated uveitis remains an important cause of ocular morbidity in children and despite some advances in the understanding of the underlying pathophysiology, sight-threatening complications are still frequently reported [53, 54]. The dearth of literature from Africa and other developing countries, reinforces the perception that immune-mediated diseases are rare or non-existent in children from this setting. Although this assumption has recently been challenged [55–58], advocacy for treatment strategies deemed too expensive, regardless of proven efficacy elsewhere, is still hindered.

Here, we have shown that the spectrum and disease characteristics associated with non- infectious uveitis are comparable in some respects to that of more developed countries but are dissimilar to reports from North Africa, where BD [24, 25] is more common. Importantly, 54.5% of our cohort presented with easily identifiable cataracts and posterior synechiae, attesting to significant delays in diagnosis. The inequitable distribution of health care resources, socioeconomic factors, health seeking behaviour and lack of expertise in key areas, contribute in varying degrees to delayed access to care in South Africa and likely resulted in late presentation to our unit [59, 60].

### Juvenile idiopathic Arthritis

Comparison with two studies from developed countries (Table 4), the large multicentre Canadian Research in Arthritis in Canadian Children emphasizing Outcomes (ReaCCH-out) study cohort and a single centre Atlanta study [27, 61], showed similarities in median age of JIA presentation, relative frequencies of poly-articular RF negative JIA subtype and ANA positivity. However, an older age at JIAU presentation and a lower frequency of oligo-articular JIA is seen in our cohort. The prevalence of JIAU here (6.5%) is also lower than reported in those studies (8.5% and 18%), as well as other developed country descriptions of up to 20% [62]. Paediatric rheumatology is a relatively new sub-speciality in South Africa, with a formal training program only established within the last 10 years. Under-recognition and lack of referral may play a role in the low frequency of JIA cases reported here. This seems to be in keeping with the few published studies from SSA [14, 20, 21, 63].

**Table 4:** Comparison of SSA and developed countries

Country	Current study: South Africa	South Africa (14)	Zambia (20)	Kenya (63)	Nigeria (21)	Canada (61)	Atlanta (27)
Single / Multi-centre	Single	Single	Single	Single	Single	Multi	Single
Total N	217	97	85	68	28	1183	287
JIA subtypes (%)							
Oligo	38.3	39	32.1	23.5	39.3	40	46
Poly RF–	19.8	30	34.6	38.2	42.8	20	24.4
Poly RF+	8.3	9	11.5	17.6	7.1	4	4.5
ERA	17.5	5	6.4	5.9	0	14	12.9
Psoriatic	7.4	0	1.3	0	0	6	3.5
SJIA	7.8	16	14.1	14.7	17.9	6	7.7
Undifferentiated	0.9	0	0	0	0	10	0.7
Uveitis (%)	6.5	4	12.8	1.5	7.1	8.5	18.2

The chronic anterior uveitis, presenting at a younger mean age, significantly in the ≤ 84-month age group (p = 0.001), also fits more developed country descriptions [62]. The majority of children with JIAU were ANA positive (78.6%). However, this is in contrast to previous studies from South Africa, where children with uveitis had polyarthritis and were ANA negative [14, 19]. Whether ancestry has an influence on these differences is uncertain. Further prospective studies to elucidate the role of sex, ancestry, JIA subtype and positive ANA, is needed.

A high percentage (21.4%), compared to the 3–7% generally described [48], developed uveitis before arthritis was diagnosed. Eleven were detected on screening and half presented with complications, 71.4% of which were cataracts. This raises further concerns of diagnostic delays in our setting. Notably, treatment was escalated to manage uveitis as arthritis was in remission.

### Idiopathic uveitis

In our study, idiopathic uveitis (36.3%) had a relatively lower frequency, reflecting the small number of referred patients likely for reasons as previously discussed. As in other descriptions, refractory disease with chronic anterior and panuveitis, complications and the need for surgery is noted. Similar severity and poorer outcome were reported in black South African children in historical studies [64–66], prior to the availability of TNFi. Here, children with refractory disease showed significant improvement in disease activity and VA on TNFi treatment.

Sarcoidosis is rare in children and may affect the uveal tract. Both children in our study responded well to systemic corticosteroid treatment and methotrexate. Escalation to TNFi was not needed here, as has been reported in other studies [67–71].

Paediatric BD is rarely reported from SSA although adult case series highlight severe skin and ocular manifestations [72, 73]. In contrast to paediatric case series from North Africa [24, 73], our patient had few recurrences, no complications and near normal vision at the last clinical assessment. HLAB51 was not done here.

Other immune mediated diseases including Vogt Koyanagi Harada syndrome, tubulointerstitial nephritis associated uveitis, uveitis with SLE and other autoinflammatory disorders were not represented in this study.

### Treatment outcome

Overall, remission on standard initial uveitis treatment (58.6%) endorses its use as first line therapy in our resource limited setting. Azathioprine and MMF were used less frequently due to gastrointestinal adverse effects and perceived lower efficacy. Neither cyclosporine nor intraocular corticosteroid injections were used in our cohort, as low evidence and side effect profile in young children were considered to outweigh the benefit [37, 46, 74, 75]. TNFi were only used in refractory cases due to availability and cost and showed good efficacy. Infliximab has been shown to be safe and efficacious in children with refractory disease [76, 77]. Here, infliximab was successfully used in 2 patients, while one switched to adalimumab due to lack of efficacy and another due to an adverse reaction to the infliximab infusion. Adalimumab was used in conjunction with methotrexate in all our patients with good effect. The small sample size limits inferences that can be made from this study but previous studies [26, 39, 40, 78] have shown good outcomes. The use of DMARDs and biologic therapy, allowed successful discontinuation of topical corticosteroid therapy in the majority of children. Further research into the efficacy of these agents in our setting is needed.

Five children with visually significant uveitic cataracts underwent lensectomies, together with pars plana vitrectomies (PPV), as is standard practice at our institution. This was due to the presence of associated vitritis or cyclitic membranes, which could only be removed by PPV.

### Selection bias

Not all children with immune-mediated uveitis may have been referred, thus community prevalence is not reflected. Children with JIA are routinely assessed for uveitis screening and may be over-represented in this sample.

### Limitations

This retrospective case file review was dependent on the availability and accuracy of the medical records reviewed.

The small sample size limits inferences that can be made from these results.

## Conclusion

Current practice seems to detect children with potentially sight-threatening non-infectious uveitis, however the low percentage of children with JIAU and the high rate of complications at presentation, raises concerns of delays in accessing appropriate healthcare. The use of DMARDs and tumour necrosis factor inhibitors in refractory cases, have improved outcomes in this cohort. Further prospective studies are needed to establish the role of associated risk factors, particularly in JIAU and the efficacy of TNFi.

## Data Availability

Privacy and confidentiality. Data was anonymised and collected in accordance with the principles of Helsinki and GCP. Data is stored in a password-protected database to which only the PI and sub-investigator has access. The data is available from the corresponding authors upon reasonable request and is stored as part of the paediatric rheumatology database and repository at the University of Cape Town.

## Abbreviations

ANA: Anti-nuclear antibody
ANA HEp2: Anti-nuclear antibody human epithelial cell indirect immuno- fluorescence test
AntiDNAse B: Anti-deoxyribonuclease B
ASOT: Anti-streptolysin O titres
BD: Behcet’s disease
DMARDs: Disease modifying anti- rheumatic drugs
ERA: Enthesitis related arthritis
FHI: Fuch’s heterochromic iridocyclitis
HIV: Human immunodeficiency virus
HLAB27: Human leukocyte antigen bB27
IL: Interleukin
ILAR: International league of associations for rheumatology
IQR: Interquartile range
ISG: International study group
JIA: Juvenile idiopathic arthritis
JIAU: Juvenile idiopathic arthritis associated uveitis
LogMAR: Log of the minimal angle of resolution
MMF: Mycophenolate mofetil
ReaCCH-out: Research in arthritis in Canadian children emphasizing outcomes
RF: Rheumatoid factor
SSA: Sub-Saharan Africa
SUN: Standard uveitis nomenclature
TB: Tuberculosis
TINU: Tubulo-interstitial nephritis associated uveitis
TNFi: Tumour necrosis factor inhibitors
VA: Visual acuity
VKH: Vogt Koyonagi Harada syndrome
